# DYNAMIC ASSOCIATIONS BETWEEN GLUCOSE AND ECOLOGICAL MOMENTARY COGNITION IN TYPE 1 DIABETES

**DOI:** 10.1093/geroni/igae098.2212

**Published:** 2024-12-31

**Authors:** Zoe Hawks, Emorie Beck, Lanee Jung, Luciana Fonseca, Martin Sliwinski, Naomi Chaytor, Laura Germine

**Affiliations:** McLean Hospital/Harvard Medical School, Belmont, Massachusetts, United States; University of California Davis, Davis, California, United States; McLean Hospital, Belmont, Massachusetts, United States; Washington State University, Spokane, Washington, United States; Pennsylvania State University, University Park, Pennsylvania, United States; Washington State University, Spokane, Washington, United States; McLean Hospital/Harvard Medical School, Belmont, Massachusetts, United States

## Abstract

Cognitive variability is associated with fall risk, cardiovascular mortality, and cognitive decline. Identifying factors that influence cognitive variability may inform evidence-based guidelines aimed at supporting cognitive and physical health across the lifespan. This goal is particularly salient in Type I Diabetes (T1D), an autoimmune condition characterized by large glucose fluctuations. T1D confers risk for neurodegeneration, and laboratory studies suggest that cognition is reduced when glucose is very low or very high. The present study leveraged advances in continuous glucose monitoring (CGM) and cognitive ecological momentary assessment (EMA) to clarify the impact of naturally-occurring glucose fluctuations on cognitive performance (sustained attention, processing speed) in 200 adults with T1D. Using hierarchical Bayesian modeling, we observed that large glucose fluctuations were associated with slower and less accurate processing speed. Glucose fluctuations were not related to sustained attention, and some individuals exhibited greater cognitive vulnerability to glucose fluctuations than others. Using data-driven lasso regression, we identified seven clinical characteristics that predicted individual differences in cognitive vulnerability to glucose fluctuations: age, time in hypoglycemia, lifetime severe hypoglycemic events, microvascular complications, glucose variability, fatigue, and characteristics (e.g. neck circumference) previously linked with weight and sleep apnea. These results establish the impact of glucose on processing speed in naturalistic environments and indicate that minimizing glucose fluctuations is important for optimizing processing speed. Beyond T1D, this work demonstrates how data from physiological sensors and EMA can be integrated to better understand individual differences in daily cognitive functioning and clinical risk.

